# Image completion algorithm of anthurium spathes based on multi-scale feature learning

**DOI:** 10.3389/fpls.2023.1281386

**Published:** 2023-12-13

**Authors:** Hongyu Wei, Jiahui Li, Wenyue Chen, Xuan Chu, Hongli Liu, Yinghui Mu, Zhiyu Ma

**Affiliations:** ^1^ College of Mechanical and Electrical Engineering, Zhongkai University of Agriculture and Engineering, Guangzhou, China; ^2^ College of Engineering, South China Agricultural University, Guangzhou, China

**Keywords:** deep learning, image completion, multi-scale, visualization, potted anthurium

## Abstract

Machine vision has been used to grade the potted anthurium plant in large-scale production recently. Images are taken to measure the number and size of anthurium spathes. However, due to the limitation of the shooting angle, the occlusion problem reduces the accuracy of measurement. It is necessary to segment the overlapping spathes and repair the incomplete ones. The traditional image completion model has good performance on missing small areas, but it is not satisfactory for missing large areas. In this article, a multi-scale fusion Recurrent Feature Reasoning (RFR) network was proposed to repair the spathe images. Unlike the traditional RFR, a multi-layer component was used in the feature reasoning module. This network can combine multi-scale features to complete the learning task and obtain more details of the spathe, which makes the network more advantageous in image completion when missing large areas of spathes. In this study, a comparison experiment between this network and the widely used image completion network was performed, and the results showed that this network performed well in all types of image completion, especially with large-area incomplete images.

## 1 Introduction

With the continuous growth of the potted Anthurium industry, automation production technology is in urgent need of improvement (GuoHua and Shuai, 2017). As an important part of anthurium automation production, grading plays a vital role in the whole production process (Pour et al., 2018; Soleimanipour et al., 2019; Soleimanipour and Chegini, 2020; Wei et al., 2021). At present, the manual grading method, which is characterized by low efficiency and accuracy, has been gradually replaced by automatic detection technology based on machine vision (Liu et al., 2023). Anthurium detection is used to measure anthurium plant height, crown width, number of flame spathes, flame spathe width, and other indicators from an image taken from above. However, when detected, the spathe is overlapped and cannot be fully visualized, which leads to a large measurement error and low classification accuracy. Therefore, it is particularly important to improve the measurement accuracy of potted anthurium by repairing the incomplete spathe after segmentation and calculating its complete contour.

Traditional image completion is mainly carried out by geometric modeling, texture matching, line fitting, and other methods (Li et al., 2013; Xia et al., 2013; Huang et al., 2014; Chan et al., 2015; Amayo et al., 2017; Iizuka et al., 2017; Li et al., 2019; Ge et al., 2022). For example, Wang et al. repaired incomplete maize leaf images by detecting and matching broken points, as well as fitting the Bezier curve of broken leaves, and then completed the segmentation of corn plants (Wang et al., 2020). Lu et al. propose a radial growth repair algorithm to repair broken roots, which takes the main root tips as the starting point and allows them to grow along the radial path. The repair accuracy of root length and diameter can reach 97.4% and 94.8%, respectively (Lu et al., 2021). Luo et al. propose a grape berry detection method based on edge image processing and geometric morphology. This method introduces edge contour search and corner detection algorithms to detect the concave position of the berry edge contour and obtain the optimal contour line. The average error of the berry size measured by this method is 2.30 mm (Luo et al., 2021). All these methods are aimed at repairing images with small missing areas but are not suitable for occluded images with large missing areas.

The development of deep learning technology has led to improved performance in image completion. (Haselmann et al., 2018; Wang et al., 2021; Zaytar and El Amrani, 2021; Belhi et al., 2023; Xiang et al., 2023; Guo and Liu, 2019; Chen et al., 2023; Mamat et al., 2023) However, it is used less in the field of agriculture. Chen et al. (2018) repaired root images of dicotyledonous and monocotyledonous plants using a convolutional neural network. Da Silva et al. (2019) reconstructs the damaged leaf parts by training a convolutional neural network model using synthetic images and then estimated the defoliation level. Silva et al. (2021) predicts the original leaf shape and estimates the leaf area based on conditional adversarial nets. Experiments show that this method can be used for leaf image completion. Zeng et al. (2022) proposed a plant point cloud completion network based on a Multi-scale Geometry-aware Transformer to solve the problem of leaf occlusion between plant canopy layers. The results show that the model is better than the current most commonly used completion networks and has a better image completion effect on plant seedlings.

At present, the deep learning algorithms for plant completion mostly include convolutional neural networks and generative adversarial networks (Geetharamani and Arun Pandian, 2019; Vaishnnave et al., 2020; Uğuz and Uysal, 2021; Yu et al., 2021; Bi and Hu, 2020; Jiao et al., 2019; Abbas et al., 2021; Zhao et al., 2021; Kumar et al., 2022; Padmanabhuni and Gera, 2022; Wong et al., 2020). Convolutional neural networks use encoders to extract potential features of the known parts of the image, and then generate the unknown parts through decoders of the image, while adding constraints to optimize repair results. The generative adversarial network is composed of two sub networks: a generator and a discriminator. The generator is used to generate relevant image data, and the discriminator is used to determine whether it is a generated image or a real image. The two networks confront each other and learn until they reach a balanced state. RFR and CRFill are two types of methods, respectively. As shown in **Table 1**, these two types of methods are not satisfactory when missing large areas, which needs to be improved.

**Table 1:** Image Completion effects of different models.

Model	0-10% missing	10-20% missing	20-30% missing	30-40% missing	40-50% missing	Original picture
Missing picture	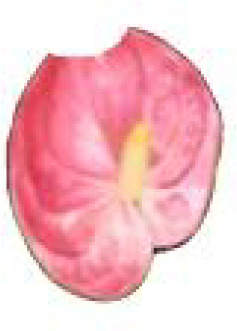	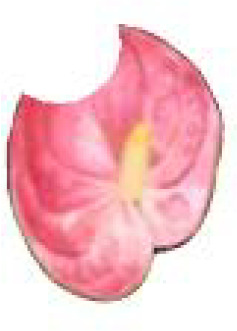	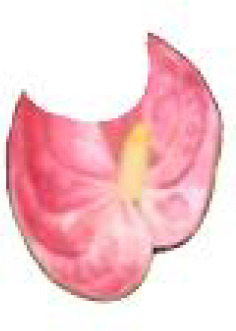	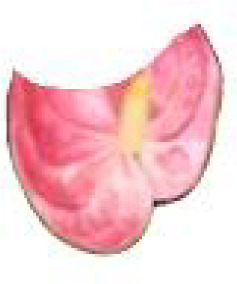	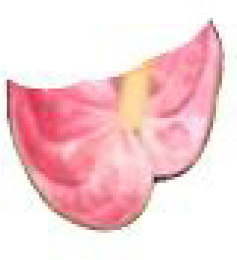	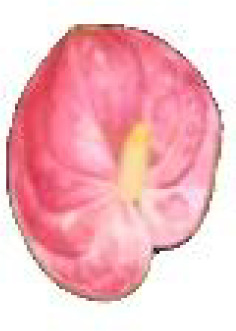
RFR	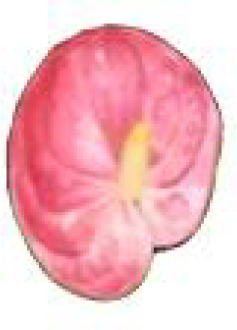	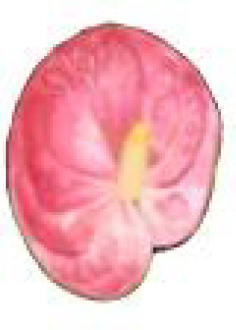	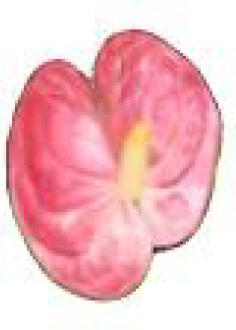	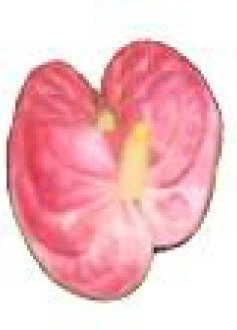	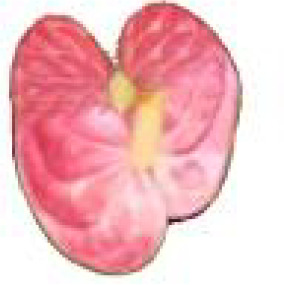	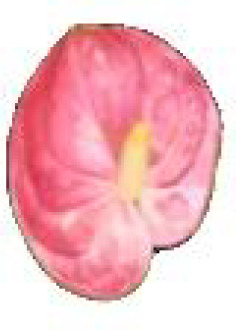
CRFill	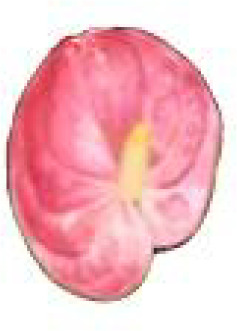	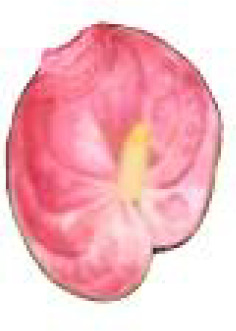	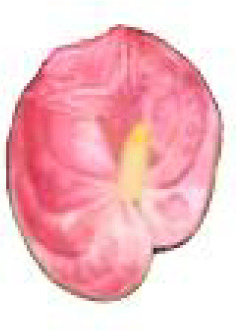	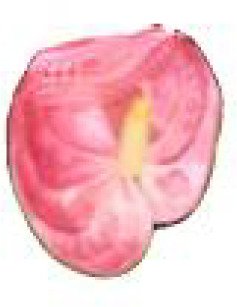	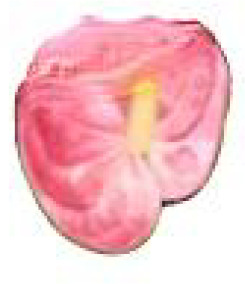	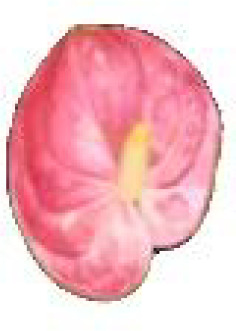

This article first analyzed the problems of existing models. Then, an improvement plan was proposed, and a comparative experiment was conducted between the improved model and the existing model. The main contributions of this article are as follows.

1. The visualization method was used to analyze the reasons for the poor performance of the RFR network in large-area missing image completion.

2. A model with strong feature learning ability was proposed, which effectively reduces the image completion error when large areas are missing.

## 2 Experiments and methods

### 2.1 Dataset establishment

Photos are taken by Azure Kinect depth camera from above in a 1.8m×1.3m×1.8m box. The distance between camera and platform is 100cm. Two 50cm long, 32w power LED light strips are installed at the same height as the camera, located on both sides of the camera 37.5cm apart, and the two light strips are at a 60° Angle to the horizontal direction. 60 pots of anthurium are used for image collection, and then the complete spathe images are extracted manually. Together with those searched from the internet, a total of 901 spathe images were collected in this study, including 726 for training and 175 for testing. Each image has a resolution of 256 x 256 and contains only one complete spathe. To improve the learning ability of the model, 726 images of the training set were scaled, rotated, and translated, and 5,320 training samples were obtained. To evaluate the performance of each model in images of different missing types and proportions, 15 groups of test samples were generated from 175 images of the test set. As shown in **Table 2**, each group was generated by 175 original images as required, and a total of 2625 images were obtained. Since the spathes are usually in the canopy layer, The occlusion of spathes is mainly caused by adjacent paths or leaves. it is found in the previous images that most of the occlusion are on one side, mainly on the root and side, and a few are on the top. Therefore, masks are randomly generated at these three parts in proportion for image training and testing.

**Table 2:** Example images of the test set.

Missing proportion Missing type	0-10%	10-20%	20-30%	30-40%	40-50%	Original picture
Top missing	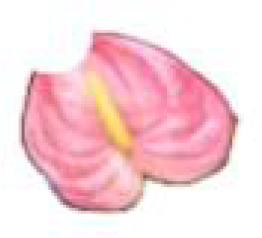	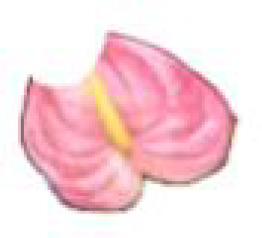	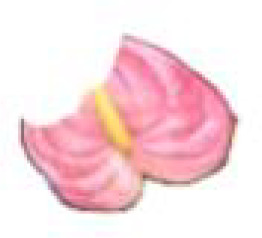	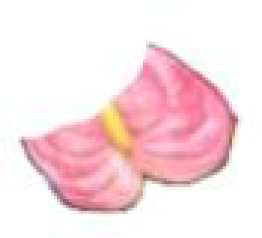	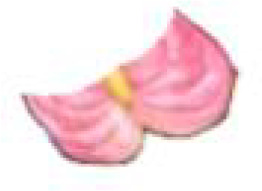	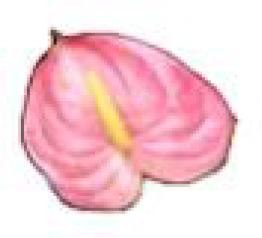
Side missing	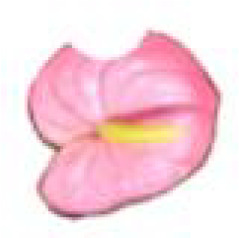	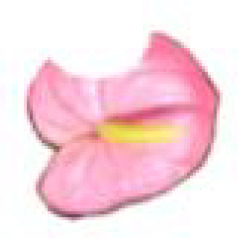	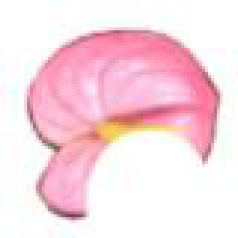	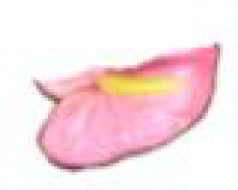	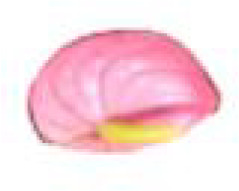	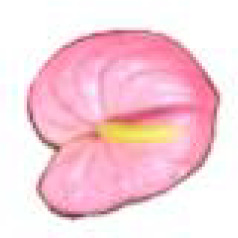
Bottom missing	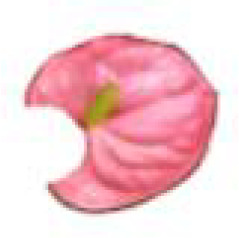	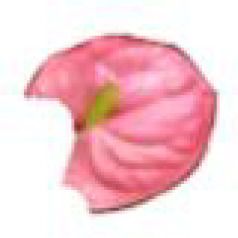	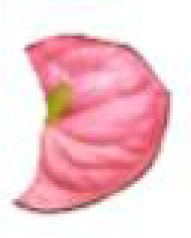	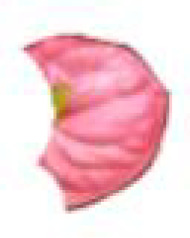	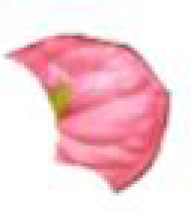	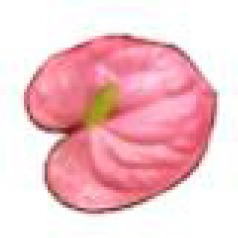

### 2.2 Visualization of recurrent feature reasoning network

**Figure 1:**
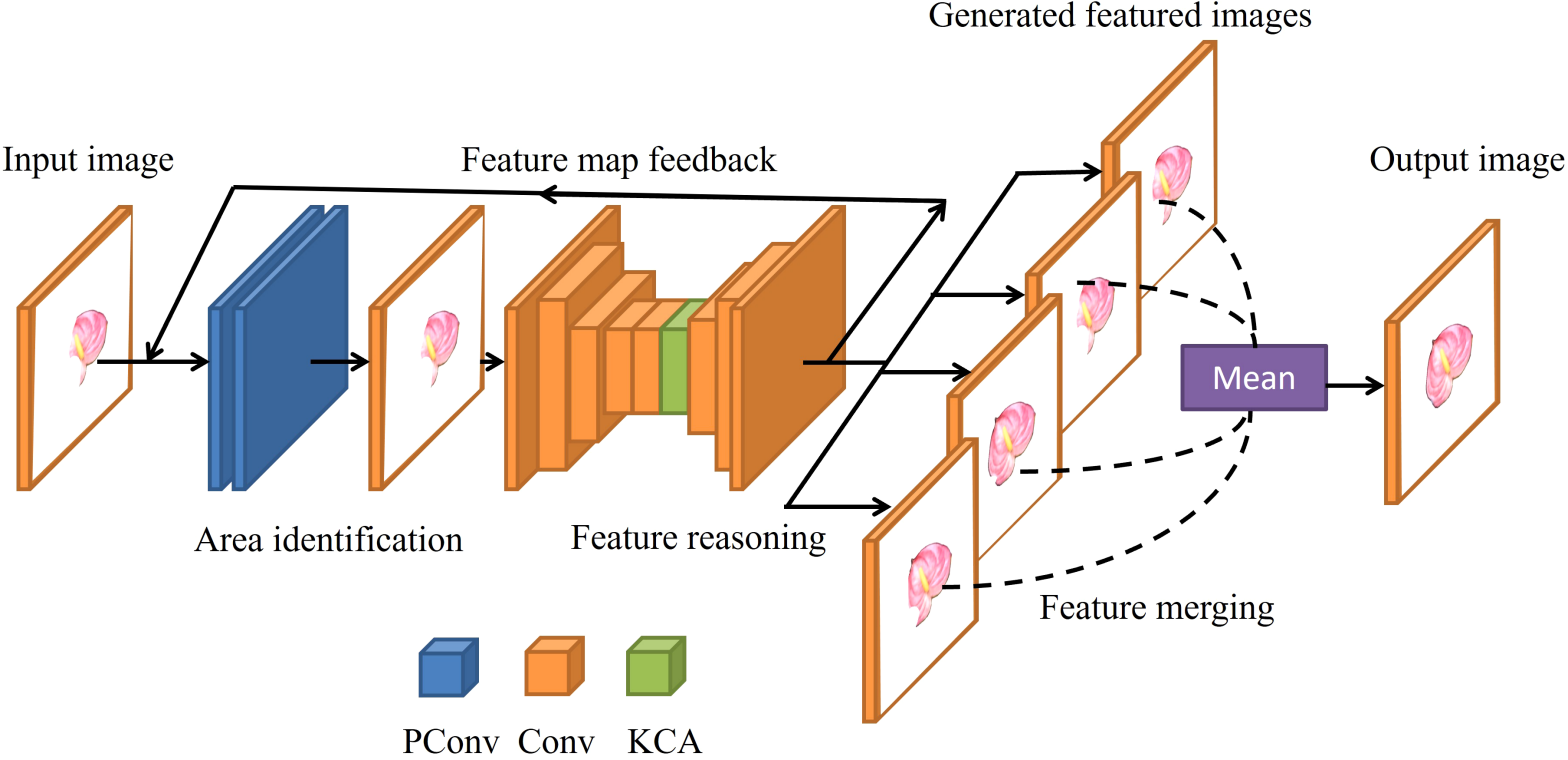
Structure of the RFR network.

RFR (Li et al., 2020) is a neural network (Szegedy et al., 2015) model for image completion, which completes images by reducing the range to be filled layer by layer, and the reuse of the parameters effectively reduces the size and running time of the model. As shown in **Figure 1**, the RFR network includes three modules: an area identification module, a feature reasoning module, and a feature merging operation. The area recognition module is used to calculate the current area that needs to be filled, and then the feature inference module fills the area. These two modules run in series and alternatively. Each run outputs the filling result of the current round. Feature merging operation fuses the features of multiple scales and outputs the final filled image.

It can be seen from the results in **Table 1** that the traditional RFR model performs well in completing missing small areas, but poorly in missing large areas. The feature reasoning module is the core of RFR, which directly affects the completion accuracy. In this study, a visual method is used to separate all feature channels of the convolution layer in the feature reasoning module, and then the visual feature map of each channel is obtained. This is helpful to determine the reason for the inadequate completion when missing large areas (Arora et al., 2014).

**Figure 2:**
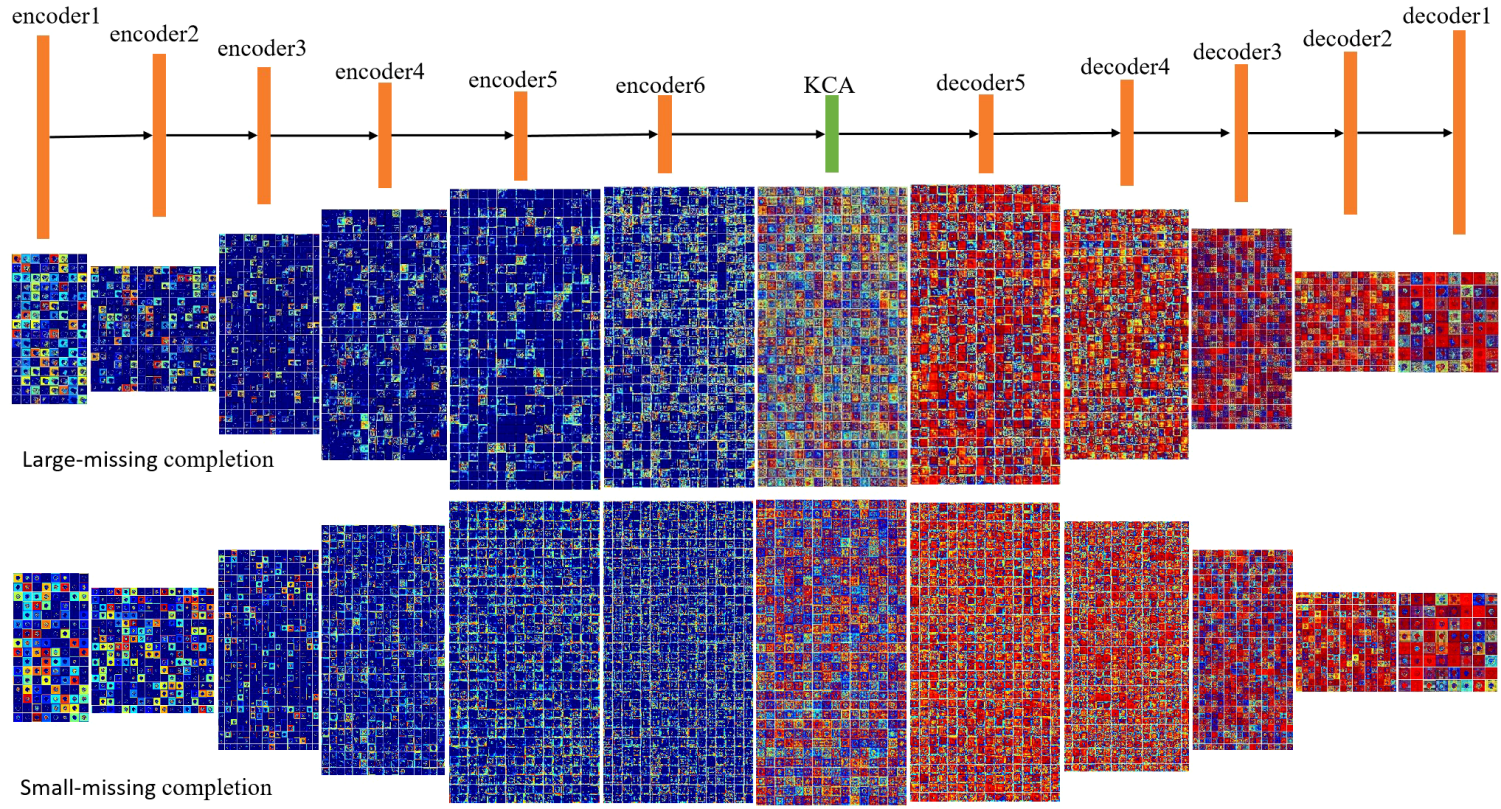
The visual results of the feature reasoning module.

**Figure 2** is the visual feature map of the coding layer and decoding layer in the feature inference module for both large and small missing cases. As can be seen from the figure that compared to small area missing images, there are more blue color blocks in large area missing images, which indicates that the semantic information extracted by the encoder in large-area missing images is relatively less. This will result in the decoder to lack enough effective information during image reconstruction, so that the weight of the red feature map is concentrated in a few feature dimensions, thus the repair result is poor.

### 2.3 Model construction

**Figure 3:**
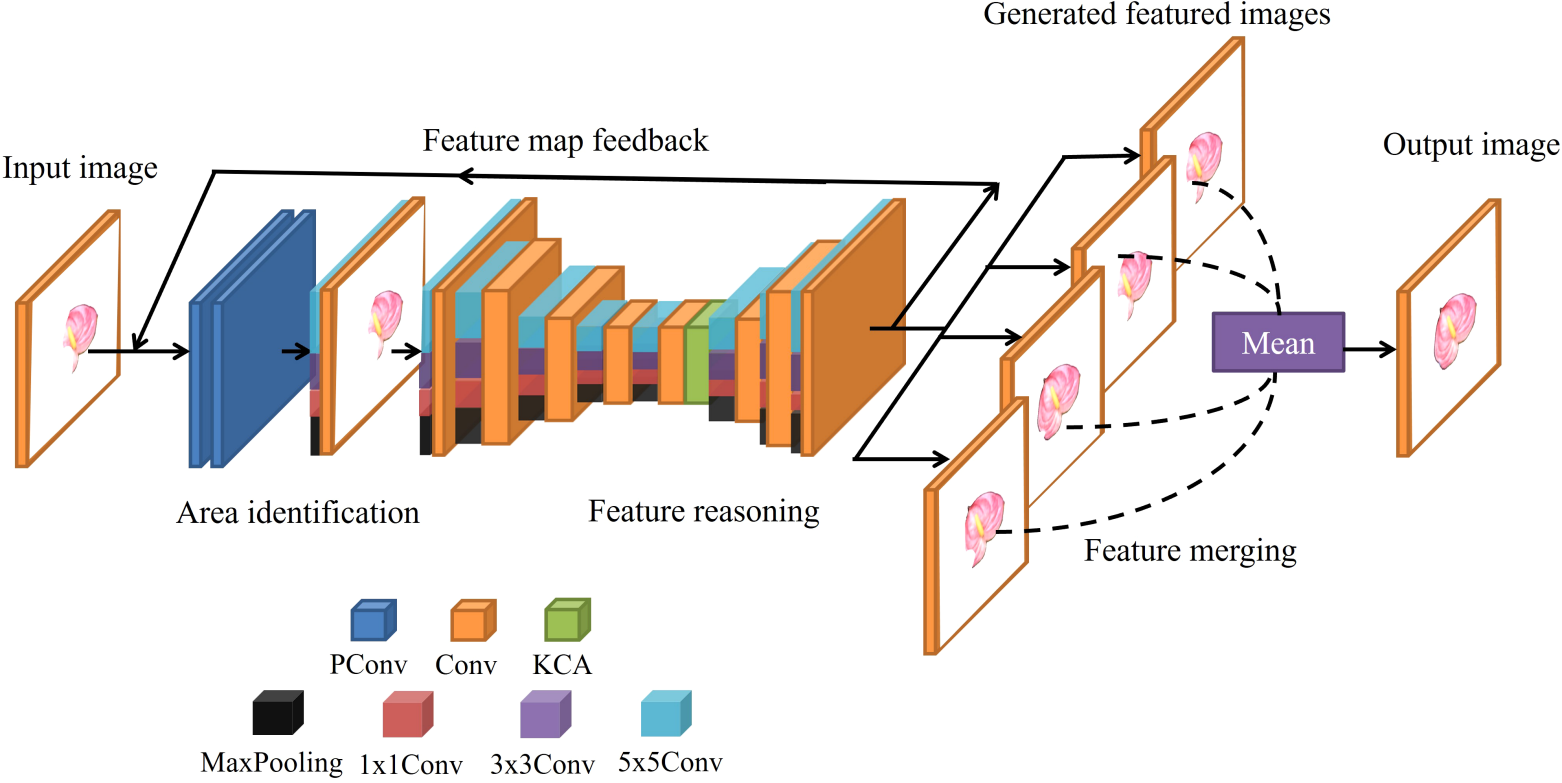
Structure of a multi-scale feature fusion RFR network.

In order to solve this problem, the Inception module is proposed to enhance the learning and reasoning ability of the network on various scale features, so as to improve the completion accuracy when large areas are missing. **Figure 3** shows the model used in this study, composed of an area identification module, a feature reasoning module, and a feature merging operation. However, different from the single layer network of RFR, a multi-layer network is used in the feature reasoning module of the model, which can fuse the features of various subsets to complete the learning task and extract richer features.

**Figure 4:**
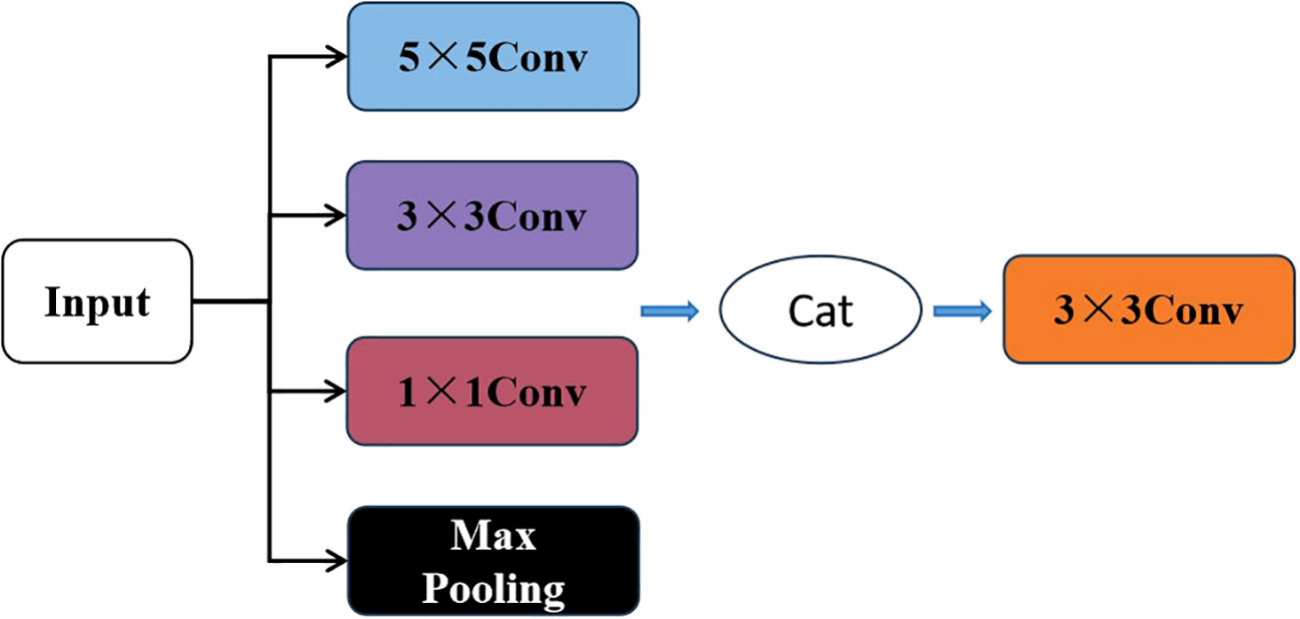
Feature reasoning module layer structure of multi-scale feature fusion RFR.

As shown in **Figure 4**, the Inception module is added to each layer of the feature reasoning module. The input image for this layer is processed through four parallel layers, and then fused by 3×3 conv. Due to the different sizes of convolutional kernels, 1×1 convolutions, 3×3 convolutions, and 5×5 convolutions have different sensory fields. More detailed features are obtained when the sensory field is smaller. At the same time, the global features are obtained by Maxpooling. This improved model can obtain not only detailed features of different scales but also global features. Therefore, the information is more comprehensive which is critical for improving the accuracy of image completion.

The calculation process is as follows:


(1) $$h_{1}^{\left(\text{i}\right)}=\text{ReLU }\left({\text{W}}_{1\text{x}1}^{\text{i}}\cdot X^{\left(\text{i}\right)}+{\text{b}}_{1}^{\left(\text{i}\right)}\right)$$

(2) $$h_{2}^{\left(\text{i}\right)}=\text{ReLU }\left({\text{W}}_{3\text{x}3}^{\text{i}}\cdot X^{\left(\text{i}\right)}+{\text{b}}_{2}^{\left(\text{i}\right)}\right)$$

(3) $$h_{3}^{\left(\text{i}\right)}=\text{ReLU }\left({\text{W}}_{5\text{x}5}^{\text{i}}\cdot X^{\left(\text{i}\right)}+{\text{b}}_{3}^{\left(\text{i}\right)}\right)$$

(4) $$h_{4}^{\left(\text{i}\right)}=\text{MaxPooling}\left(X^{\left(\text{i}\right)}\right)$$

(5) $$h^{\left(\text{i}\right)}=\text{LeakyReLU}\left({\text{W}}_{3\text{x}3}^{\text{i}}\cdot \text{Concat}\left(h_{1}^{\left(\text{i}\right)},h_{2}^{\left(\text{i}\right)},h_{3}^{\left(\text{i}\right)},h_{4}^{\left(\text{i}\right)}\right)\right)$$

where ${\text{h}}_{1}^{\left(\text{i}\right)},{\text{h}}_{2}^{\left(\text{i}\right)},{\text{h}}_{3}^{\left(\text{i}\right)},{\text{h}}_{4}^{\left(\text{i}\right)}$ represent the output of 1×1 convolutions, 3×3 convolutions, 5×5 convolutions, and Max Pooling, respectively, and ${\text{h}}^{\left(\text{i}\right)}$ represents the result of concatenation and convolution processing of the four components. ${\text{W}}_{1\text{x}1}^{\text{i}}$ represents the weight matrix of the 1×1 convolution of layer i, and similarly, ${\text{W}}_{3\text{x}3}^{\text{i}},{\text{ W}}_{5\text{x}5}^{\text{i}}$ represent the weight matrix of 3×3 convolution and 5×5 convolution, respectively. ${\text{X}}^{\left(\text{i}\right)}$ is the output feature map of the previous layer network. ${\text{b}}_{1}^{\left(\text{i}\right)},{\text{ b}}_{2}^{\left(\text{i}\right)},{\text{ b}}_{3}^{\left(\text{i}\right)}$ represent the bias terms of the 1×1 convolution, 3×3 convolution, and 5×5 convolution, respectively. ReLU and LeakyReLU represent activation functions.

**Figure 5:**
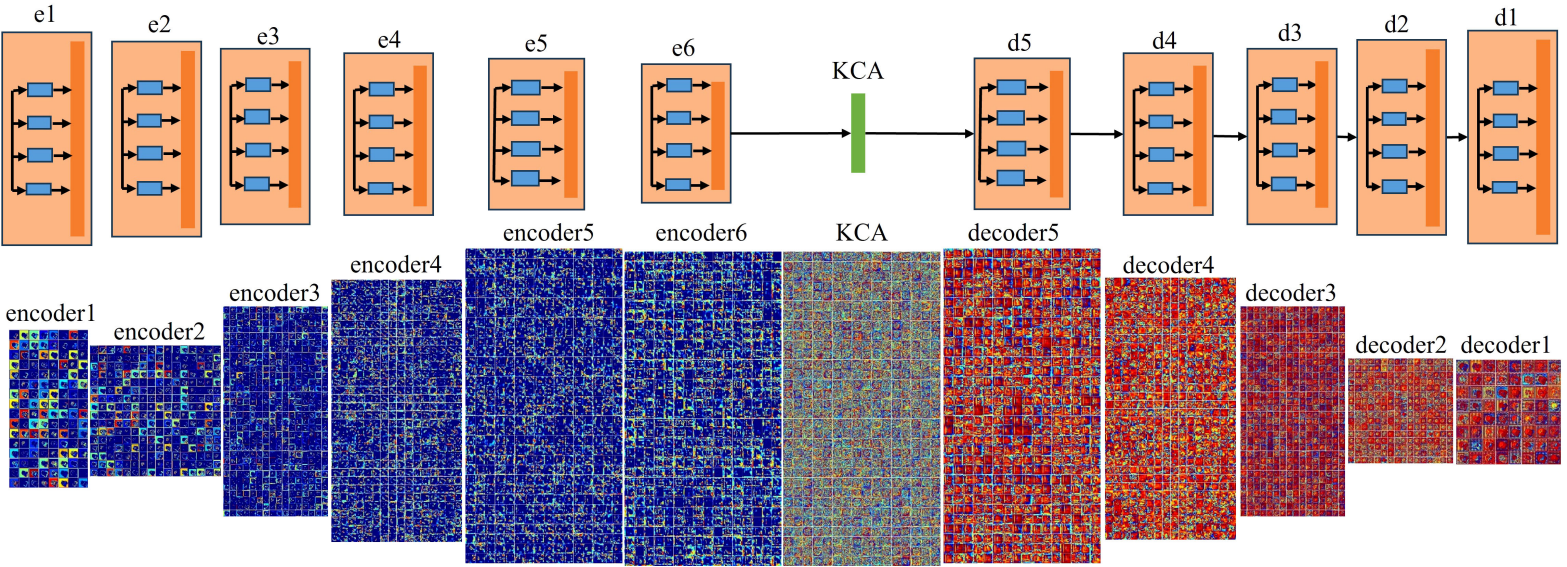
The visual results of the improved feature reasoning module.

**Figure 5** shows the visual results of the improved feature reasoning module on the above large area missing image. Compared with RFR, this model has richer feature information in both the encoding and decoding processes, which also indicates that this model effectively improves the learning ability of the feature reasoning module.

### 2.4 Model training

Transfer learning was used to speed up the convergence, and the Adam optimizer was used, with a learning rate of $2\times 10^{4}$ , a batch size of 4, and 120,000 as the number of iterations. The multi-scale image completion network was trained by a joint loss function consisting of the content loss of the completed part, the content loss of the whole spathe, and the perceptual and style losses, to improve the consistency of the completed image and the real image. The expression of the loss function is as follows:


(6) $$L_{sum}=\lambda_{hole}L_{hole}+\lambda_{valid}L_{valid}+\lambda_{\text{perceptual}}L_{\text{perceptual}}+\lambda_{style}L_{style}$$

where, ${\text{L}}_{\text{sum}}$ is the loss function, $L_{hole}$ is the content loss of the completed part, ${\text{L}}_{\text{valid}}$ is the content loss of the whole spathe, ${\text{L}}_{\text{perceptual}}$ is the perceptual loss, and ${\text{L}}_{\text{style}}$ is the style loss. In this article, the loss function coefficients are set as ${\text{λ}}_{\text{hole}}=1,{\text{ λ}}_{\text{valid}}=6,{\text{ λ}}_{\text{perceptual}}=0.05,{\text{ λ}}_{\text{style}}=120$ . The random mask algorithm was used to automatically generate missing images during training. Two types of comparison experiments were designed according to the proportion and type of the missing, and the completion results were compared with Four widely used models CRFill, RFR, CTSDG and WaveFill. CTSDG uses a bi-gated feature fusion (Bi-GFF) module to integrate reconstructed structure and texture maps to enhance their consistency. WaveFill is based on wavelet transform, breaking the image into multiple frequency bands and filling in the missing areas in each band separately.

### 2.5 Evaluation indicator

In this article, qualitative and quantitative methods are used to evaluate the repair result. The quantitative evaluation mainly shows the degree of improvement in image completion compared with other models, which needs to be analyzed in combination with the results of qualitative analysis.

**Figure 6:**
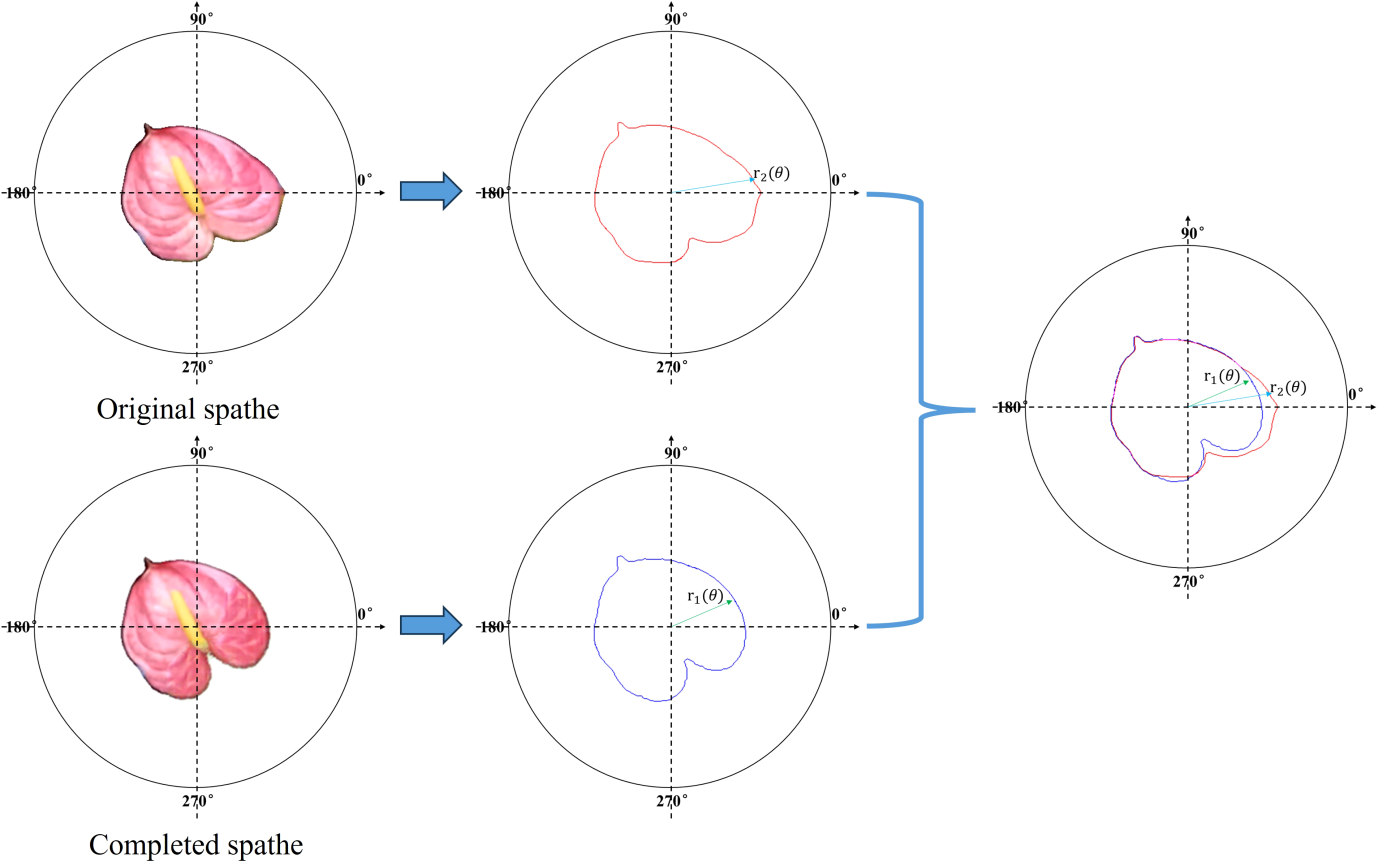
Accuracy evaluation of spathe image completion.


To evaluate the completion accuracy of the model, the following polar coordinate system was established on the surface of the spathe. As shown in **Figure 6**, assuming that the quality of each pixel in the image is uniform, the centroid of the spathe is taken as the pole. Horizontally to the right indicates 0° of the polar axis, and counterclockwise is the positive direction of the angle. The unit of the axes in polar coordinate system are pixels. The contours extraction algorithm is used, and the contours of the completed spathe and real spathe are $r_{1}\left(\theta \right)$ and $r_{2}\left(\theta \right)$ , respectively. Mean square error(MSE) is a commonly used index to measure the difference between the predicted value and the actual observed value, and it can well represent the degree of fitting between the predicted contour and the real contour. The calculation formula is as follows:

(7) $$\text{MSE}=\;\frac{1}{2\pi }\int_{0}^{2\pi }{\left(r_{1}\left(\theta \right)-r_{2}\left(\theta \right)\right)}^{2}\text{d}\theta$$

where, θ is the polar angle of the polar coordinate system, and r(θ) is the distance from the centroid to the contour edge when the polar angle is θ . A smaller mean squared error correlates with higher measurement accuracy.

## 3 Results and discussion

### 3.1 Qualitative evaluation

To qualitatively evaluate the completion effect of the model in this study, repair experiments were carried out on the images of 15 groups of test sets, and the results are shown in **Tables 3**–**5**. It can be seen from the completion results that CRFill has the worst performance of the three types and can hardly repair images with large missing areas. RFR is prone to errors, and the results are variable. The other three models can complete a similar spathe profile. However, compared with the model presented in this article, CTSDG and WaveFill cannot accurately complete the detailed features in images with large missing areas, and the total deviation is large. The model in this article adds the Inception module, which utilizes additional reasoning features in large-area completion. Even when the image is 40-50% missing, the model still demonstrates good completion ability, which is very important for phenotype detection.

**Table 3:** Comparison of top missing image completion results.

	0-10%	10-20%	20-30%	30-40%	40-50%
Original	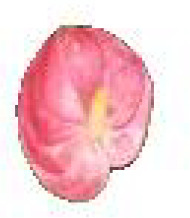	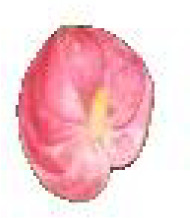	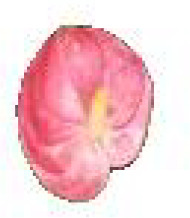	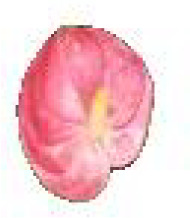	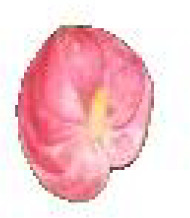
Missing	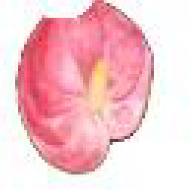	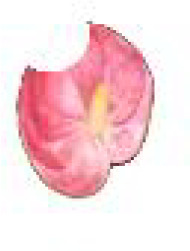	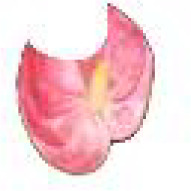	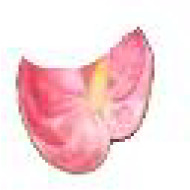	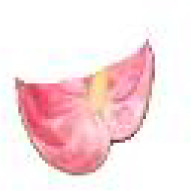
CRFill	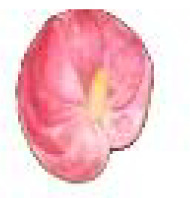	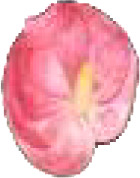	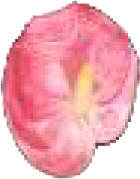	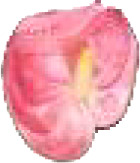	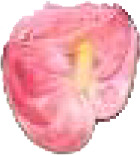
CTSDG	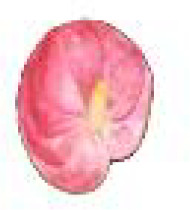	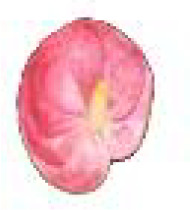	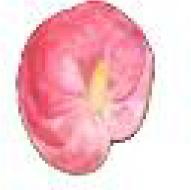	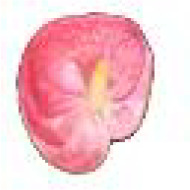	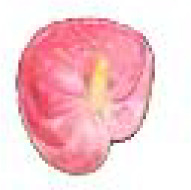
WaveFill	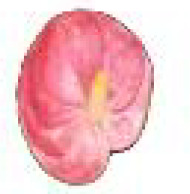	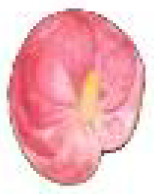	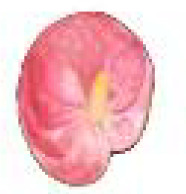	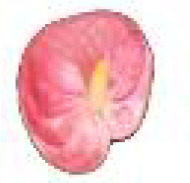	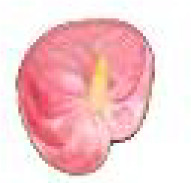
RFR	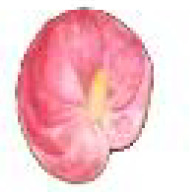	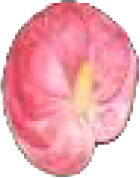	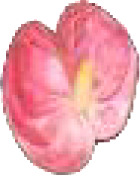	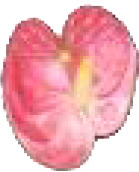	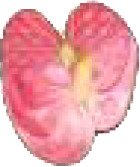
Ours	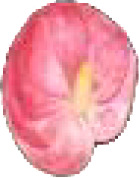	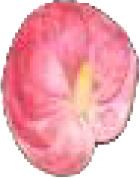	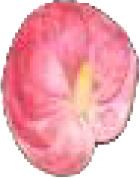	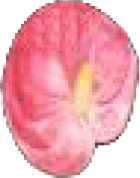	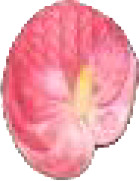

**Table 4:** Comparison of side missing image completion results.

	0-10%	10-20%	20-30%	30-40%	40-50%
Original	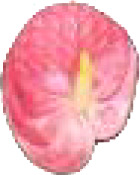	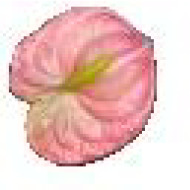	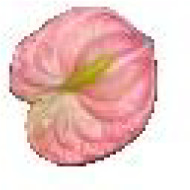	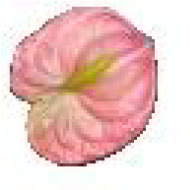	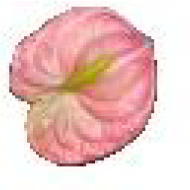
Missing	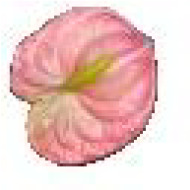	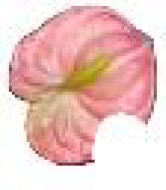	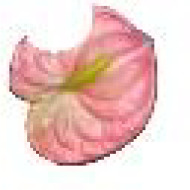	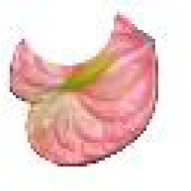	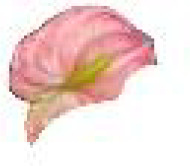
CRFill	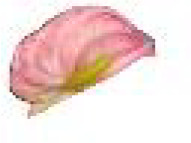	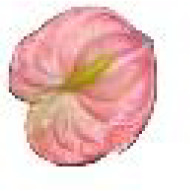	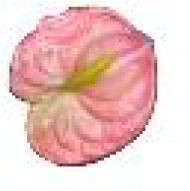	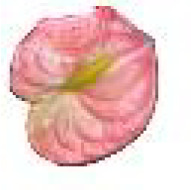	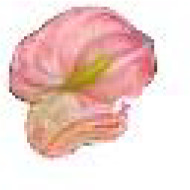
CTSDG	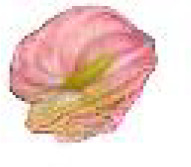	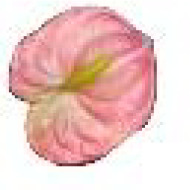	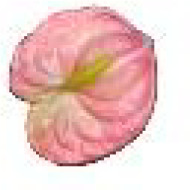	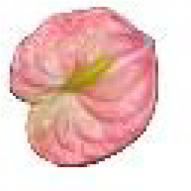	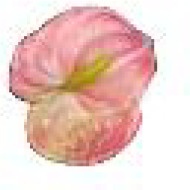
WaveFill	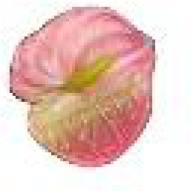	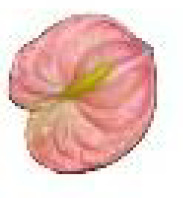	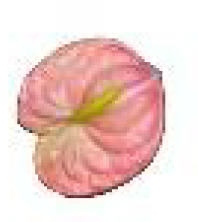	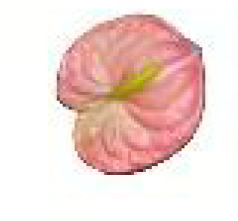	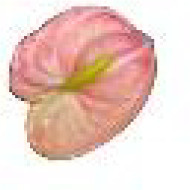
RFR	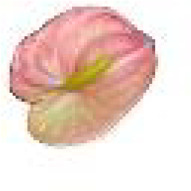	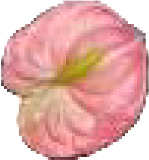	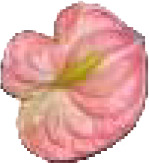	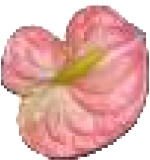	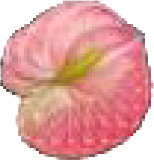
Ours	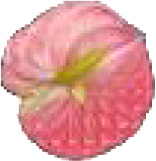	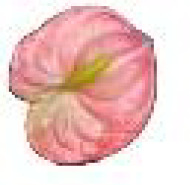	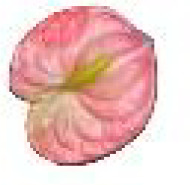	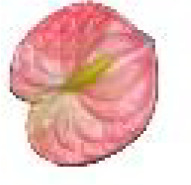	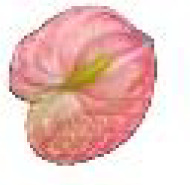

**Table 5:** Comparison of bottom missing image completion results.

	0-10%	10-20%	20-30%	30-40%	40-50%
Original	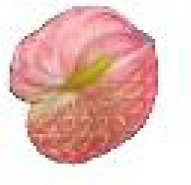	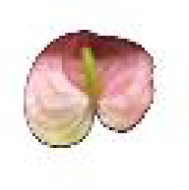	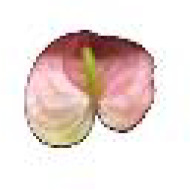	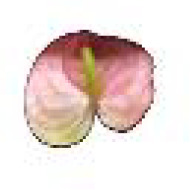	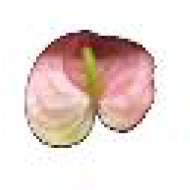
Missing	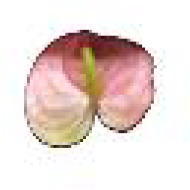	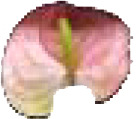	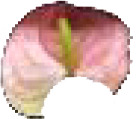	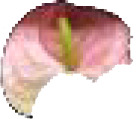	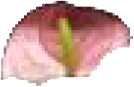
CRFill	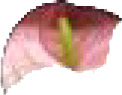	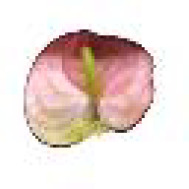	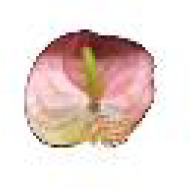	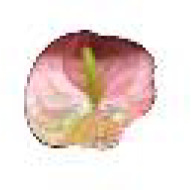	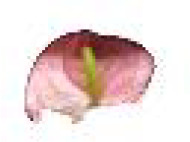
CTSDG	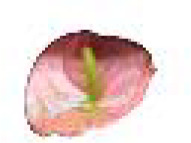	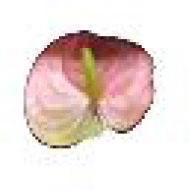	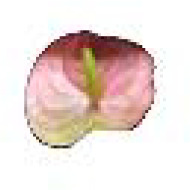	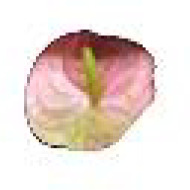	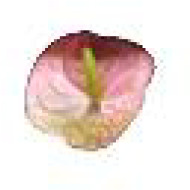
WaveFill	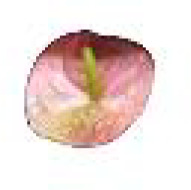	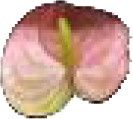	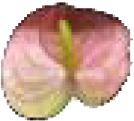	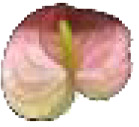	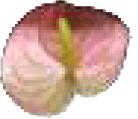
RFR	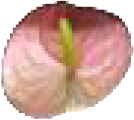	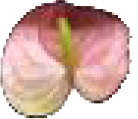	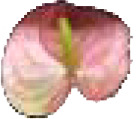	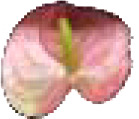	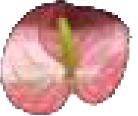
Ours	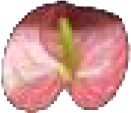	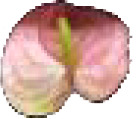	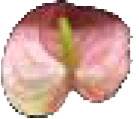	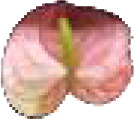	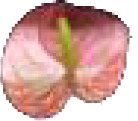

### 3.2 Quantitative evaluation

#### 3.2.1 Influence of incomplete type on image completion results

**Figure 7:**
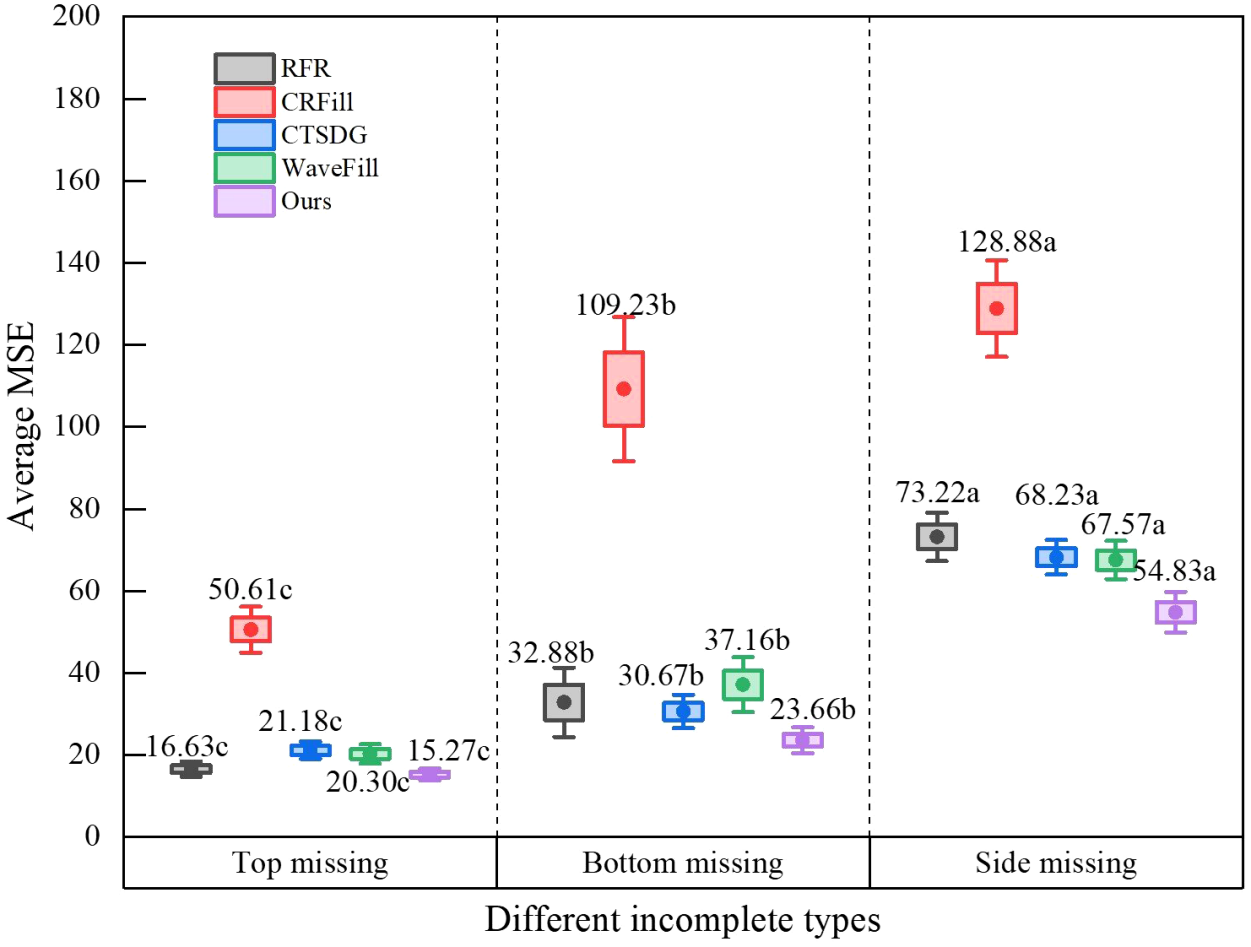
Average MSE of different incomplete types.

**Figure 7** shows the image completion accuracy of each model for different incomplete types. It can be seen that CRFill performs poorly in all types of image completion and differs significantly from others. For the top-missing type, other models perform well with a mean square error between 15.27 and 21.18. Meanwhile, for the side-missing and bottom-missing types, the mean square error of image completion increases, but the values of our model are still the smallest among all models, which are 23.66 and 54.83 respectively. Among the five types, the error in the bottom-missing type is large, which is due to the significant individual variances at the bottom of the spathe and make it difficult to complete. However, of all the types, the model in this study has the best performance and the accuracy is higher than other models.

#### 3.2.2 Influence of incomplete proportions on image completion results

**Figure 8:**
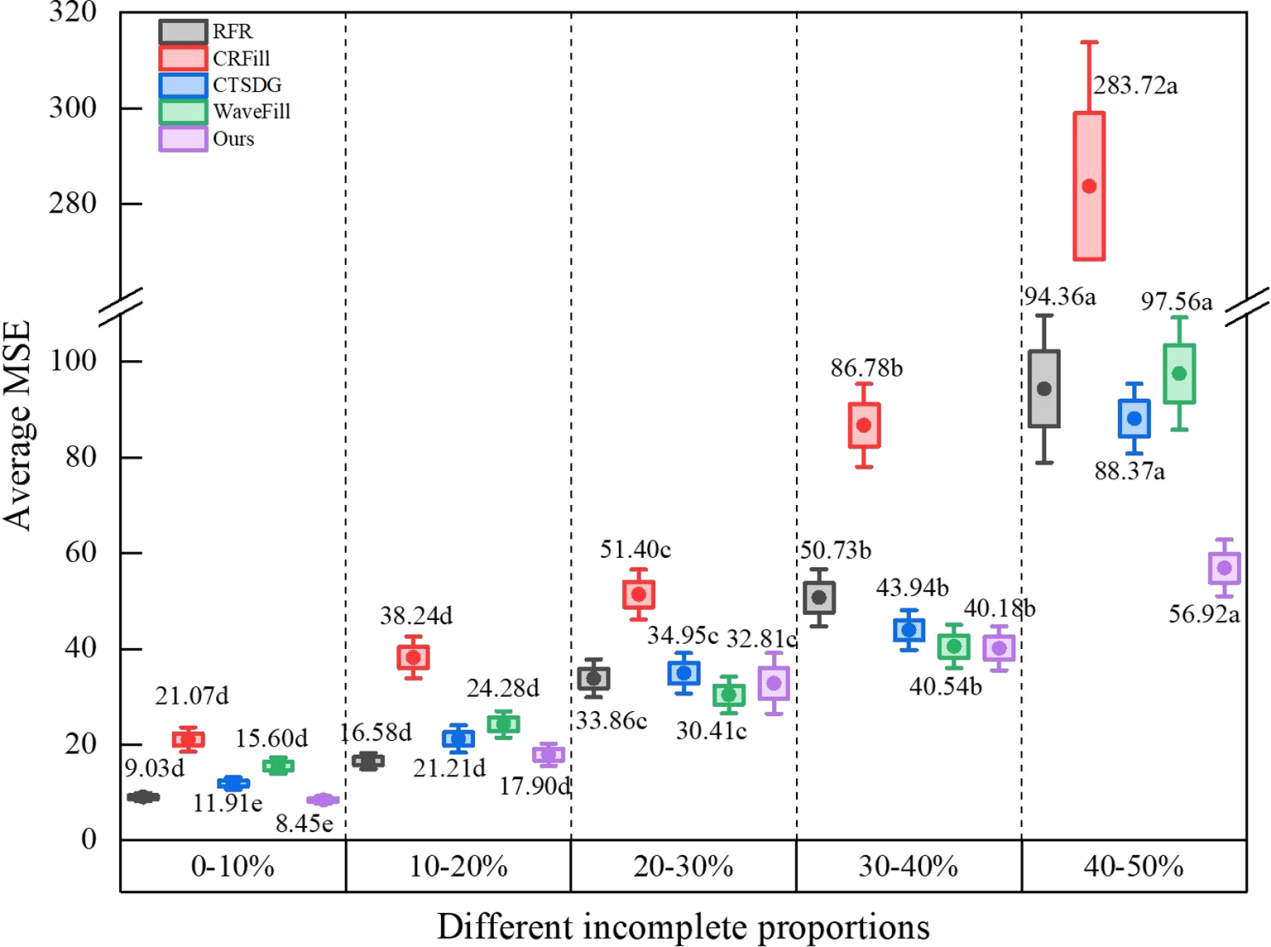
Average MSE of different incomplete proportions.

**Figure 8** shows the image completion accuracy of each model for different incomplete proportions. The incomplete proportion has a significant impact on the completion accuracy. Similarly, aside from CRFill, the completion accuracy of the other models is adequate when the incomplete proportion is less than 10%. With an increase in the incomplete proportion, the average MSE gradually increases. When the incomplete proportion reaches 40-50%, the average MSE significantly increases. This is because when a large proportion is missing, the number of features used for reasoning is reduced. It can also be seen from the results that when the incomplete proportion is less than 40%, the average MSE of the model in this study has little difference from RFR, CTSDG, and WaveFill. However, when the incomplete proportion reaches 40-50%, the model shows a significant advantage, approaching half of the error of the others. According to the qualitative evaluation results in 3.2.1, there are obvious errors in the repair results of other models when the incomplete proportion is 40%-50%, while the result of the improved model is in good agreement with the original image. Therefore, this error is considered acceptable.

It can be seen from the results of comparative experiments that Inception module combines different convolution layers in parallel and connects the result matrices processed by different convolution layers together to form a deeper matrix in depth dimension. It can aggregate visual information of different sizes and reduce the dimensionality of larger matrices to extract features of different scales. Therefore, the information obtained by the improved model is more abundant, and the accuracy of image completion is effectively improved.

## 4 Conclusion

This study analyzed the reasons for the low completion accuracy of the RFR model in large-area missing images by visual methods. The Inception module was proposed to improve the feature reasoning module of the RFR model, which further improved the feature learning ability. The improved model could obtain not only the detailed features of different scales, but also the global features, which perform well. In missing type comparison experiment with existing widely used models, it can be seen that the top-missing type has the best results, followed by side-missing type, and bottom-missing type has the largest repair error due to significant individual differences. However, no matter what kind of missing type, the model presented in this article has obvious advantages. In the comparative experiments of different missing parts, it was found that the repair error of each model increased with the increase of missing proportion. When the incomplete ratio reaches 40-50%, the error of this model is only half that of others. This shows that this model performs best regardless of the type and proportion of missing images, and its repair accuracy is significantly higher than other models, which is crucial for improving the measurement accuracy of potted anthurium. Although the method in this article integrates features of different scales, it is still based on two-dimensional images, ignoring the influence of the tilt Angle of spathes. If depth information can be introduced to repair images in three-dimensional space in the future, the repair accuracy can be further improved.

## Data availability statement

The raw data supporting the conclusions of this article will be made available by the authors, without undue reservation.

## Author contributions

HW: Conceptualization, Formal Analysis, Methodology, Writing – original draft, Writing – review & editing. JL: Data curation, Formal Analysis, Writing – original draft, Writing – review & editing. WC: Data curation, Formal Analysis, Writing – review & editing. XC: Funding acquisition, Writing – review & editing. HL: Writing – original draft, Writing – review & editing. YM: Conceptualization, Methodology, Writing – review & editing. ZM: Conceptualization, Funding acquisition, Methodology, Writing – review & editing.
